# Creating a web-based electronic tool to aid tuberculosis (TB) cluster investigation: data integration in TB surveillance activities in the United Kingdom, 2013 to 2016

**DOI:** 10.2807/1560-7917.ES.2018.23.44.1700794

**Published:** 2018-11-01

**Authors:** Jennifer A Davidson, Laura F Anderson, Victoria Adebisi, Leonardo de Jongh, Andy Burkitt, Maeve K Lalor

**Affiliations:** 1 Tuberculosis Section, National Infection Service, Public Health England, London, United Kingdom; 2 Global TB, World Health Organization, Geneva, Switzerland; 3 Software Development Unit, National Infection Service, Public Health England, London, United Kingdom; 4 Field Epidemiology Services, National Infection Service, Public Health England, Newcastle, United Kingdom; 5 Institute for Global Health, University College London, London, United Kingdom

**Keywords:** surveillance, automated surveillance, tuberculosis, genotyping

## Abstract

Molecular technology to identify relatedness between *Mycobacterium tuberculosis* complex isolates, representative of possible tuberculosis (TB) transmission between individuals, continues to evolve. At the same time, tools to utilise this information for public health action to improve TB control should also be implemented. Public Health England developed the Strain Typing Module (STM) as an integral part of the web-based surveillance system used in the United Kingdom following the roll-out of prospective 24 loci mycobacterial interspersed repetitive unit-variable number tandem repeat (MIRU-VNTR) strain typing. The creation of such a system required data integration and linkage, bringing together laboratory results and patient notification information. The STM facilitated widespread access to patient strain typing and clustering results for the public health community working in TB control. In addition, the system provided a log of cluster review and investigation decision making and results. Automated real-time data linkage between laboratory and notification data are essential to allow routine use of genotyping results in TB surveillance and control. Outputs must be accessible by those working in TB control at a local level to have any impact in ongoing public health activity.

## Introduction

Tuberculosis (TB) incidence in the United Kingdom (UK) increased during the early 2000s from 12.3 per 100,000 in 2000 to 15.1 per 100,000 in 2005 [1]. Following this, recommendations for improvements in TB control were made in the Chief Medical Officer’s Action Plan for TB [2]. This included improved TB surveillance activity through the implementation of routine prospective molecular typing. In response, in 2010, the National TB Strain Typing Service (TB STS) was established by Public Health England [3] to prospectively identify and, where necessary, investigate 24 loci mycobacterial interspersed repetitive unit-variable number tandem repeat (MIRU-VNTR) strain typing clusters [4].

The routine, prospective and universal use of molecular characterisation techniques, such as MIRU-VNTR strain typing, have multiple benefits in clinical care and public health as previously described [5]. One of which is the role in identifying TB transmission, with an aim to interrupt it. This can, firstly, be achieved by confirming or refuting suspected transmission using MIRU-VNTR, secondly, by identifying MIRU-VNTR clusters where there had previously been no suspicion of transmission.

To efficiently use MIRU-VNTR strain typing results for public health action in the UK, the data collected in routine TB notification and culture results in laboratory isolate records were linked. This was achieved within a custom-designed Strain Typing Module (STM) embedded in the current web-based national surveillance system, the Enhanced Tuberculosis Surveillance System (ETS). The STM was therefore able to provide one central database and a user-friendly real-time view of all MIRU-VNTR clusters along with the associated patient information. These data sources combined are required to review clusters and investigate whether public health action was required, beyond routine contact tracing [4].

In this paper we describe the process of linking TB notification and laboratory isolate records and outline the structure and features of the STM. In addition, we describe its use in the systematic detection of clusters, their review and recording the management of outbreaks and investigations in the UK. We hope that reflecting on our experience of integrating genotyping into TB surveillance for routine use will provide insights for other countries planning to make efficient use of molecular TB data. In addition, within the UK we can build upon our experience to develop an improved system for the roll-out of whole genome sequencing (WGS).

## Methods

### Dataflow and record linkage

A simple and easily maintained dataflow setup which utilises multiple data sources was essential to ensure that information was readily available and in a suitable format for users, in this instance public health professionals working in TB control.

In the UK, all *Mycobacterium* isolates cultured in local laboratories were sent to one of the reference laboratories for speciation, drug susceptibility testing (DST) and molecular typing (24 loci MIRU-VNTR strain typing between 2010 to 2016; strain typing in the UK is being gradually phased out starting from 2016 to be replaced with WGS). Mechanisms were developed to extract these results (for all culture-positive *Mycobacterium* isolates) along with patient identifiers from the *Mycobacterium* reference laboratory information management systems (LIMS) with output sent to Public Health England’s national TB Surveillance Unit.

**Figure 1:**
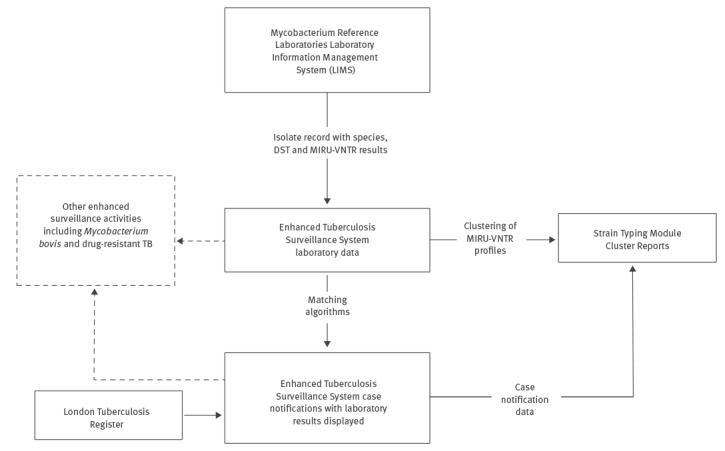
Tuberculosis data flow and linkage for cluster review and investigation, United Kingdom, 2013–2016

DST: drug susceptibility testing; MIRU-VNTR: mycobacterial interspersed repetitive unit-variable number tandem repeat; TB: tuberculosis.

**Figure 2:**
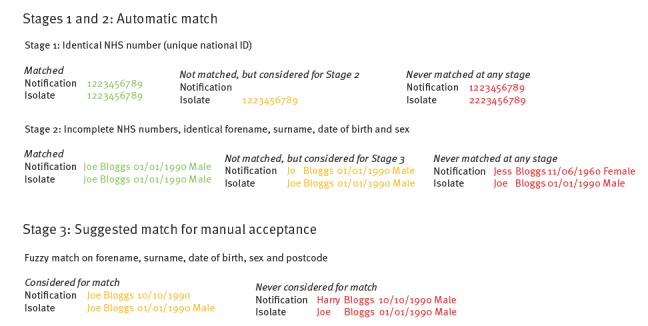
Algorithm to link tuberculosis laboratory and case data, United Kingdom, 2013–2016

ID: identification number; NHS: National Health Service.

At the time of implementing the TB STS, which prescribed action to be taken based on the findings of strain typing data, there was no mechanism to easily incorporate strain typing results into the electronic surveillance system on a routine basis [6]. Therefore, to facilitate routine cluster review and investigation, designated cluster investigators extracted the relevant demographic, clinical and risk factor data from ETS for clustered cases based on MIRU-VNTR Microsoft Excel reports received from the reference laboratories. They manually linked these data in a Microsoft Access database. This resulted in significant manual manipulation of data, which was laborious and time-consuming and can lead to the introduction of mistakes. In order to streamline the process and reduce processing time, an automated process was set up to import laboratory results directly into ETS where they could be linked with patient data (Figure 1). Automated record linkage was implemented using an algorithm that matches patient identifiers from isolate records with those of notifications using two matching methods: (i) by automatically assigning the laboratory result to a notification through deterministic matching or (ii) by displaying the laboratory result as a possible match for a notification using probabilistic matching (Figure 2).

For all stages (1–3) matches are only considered if the notification and specimen date are within 365 days of each other. This is to avoid matching isolates to the wrong episode of tuberculosis if a patient has multiple episodes.

### Data manipulation and algorithms

**Figure 3:**
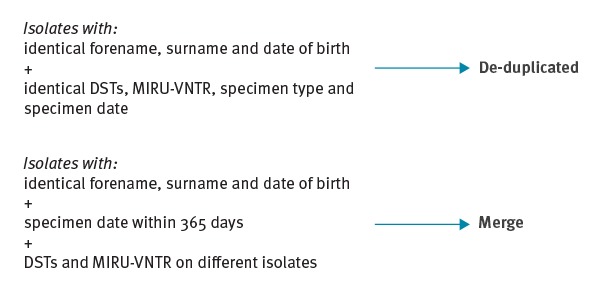
De-duplication and merging of tuberculosis laboratory results in the Strain Typing Module, United Kingdom, 2013–2016

DST: drug susceptibility testing; MIRU-VNTR: mycobacterial interspersed repetitive unit-variable number tandem repeat.

The STM was integrated directly into ETS (Figure 1) as a module to display clusters of patients’ within specific geographies. To ensure the display in the STM was useful for public health teams reviewing clusters, an algorithm was developed to process the isolate data and return clusters with one summary result per patient’s TB episode. This process ensured that all isolates belonging to the same patient (identified by identical forename, surname and date of birth) and sampled within 365 days of each other were de-duplicated and merged together to create one record per patient (Figure 3).

A further algorithm was then implemented to compare these merged and de-duplicated isolates, representative of a patient’s TB episode, identifying patients with indistinguishable strain types which therefore comprised molecular clusters. The definition used for a cluster in the UK was two or more patients with indistinguishable 24 loci strains, with at least one having a complete 24 loci MIRU VNTR profile; additional strain types in the cluster may each have one missing locus [4]. Each cluster was assigned a name within the system, the nomenclature for naming clusters being a letter which denotes the phylotype of the strain (A = East African Indian, B = Beijing, C = Central Asian, E = Euro-American, F = *Mycobacterium africanum*, V = *M. bovis*, X = multiple global lineages, U = no global lineage) followed by a four digit number assigned in sequential order (for example B1001, then B1002).

## Results

### Data collected in the system

Between 2010 and 2015, the ETS laboratory database held records for 42,148 isolates which the system de-duplicated as representative of 28,741 TB patient episodes, of which 23,646 (82.3%) had a strain typing result. These individuals belonged to 2,701 different molecular clusters, which were displayed in cluster reports in the STM. In addition, 89 cases (either not culture-confirmed or not typed to 24 loci) were manually added to clusters in the STM based on epidemiological intelligence and information gathered through cluster investigation.

Between 2013 (when daily import of new laboratory results into ETS commenced) and 2015 (the last full calendar year of strain typing use in the UK), the median time between specimen receipt in the reference laboratory and the MIRU-VNTR results being available to view in ETS was 2.3 months (70.9 days; interquartile range (IQR): 46.8–123.8).

### Features and functions of the Strain Typing Module

**Figure 4:**
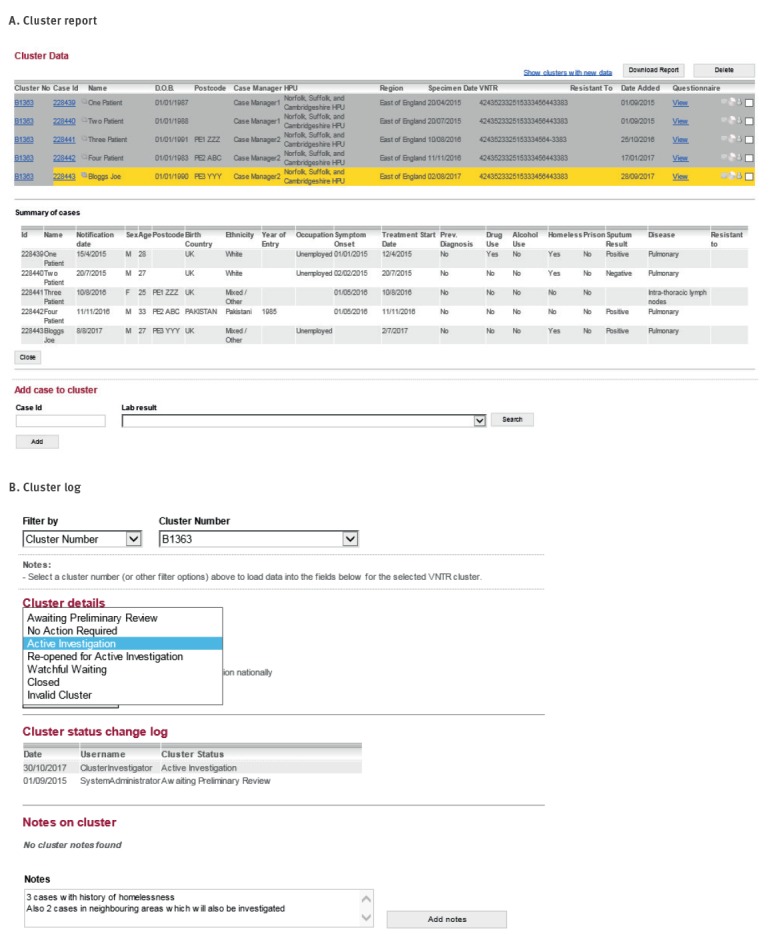
Features and functionality of the Strain Typing Module, United Kingdom, 2013–2016

DOB: date of birth; F: female; HPU: Health Protection Unit; M: male; No.: number; UK: United Kingdom; VNTR: variable number tandem repeat.

**Figure 5:**
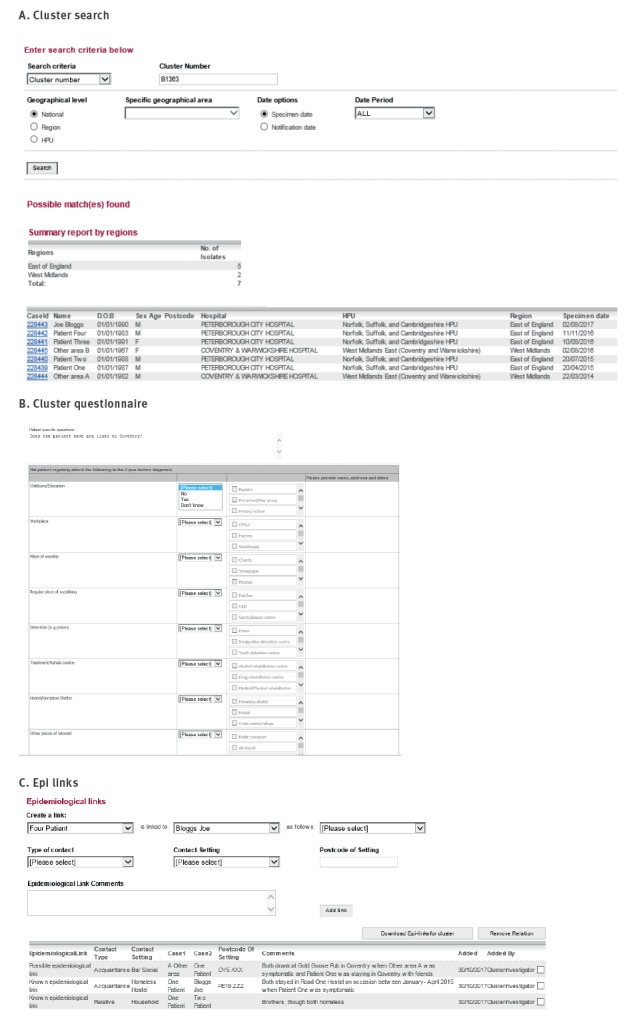
Features and functionality of the Strain Typing Module, United Kingdom, 2013–2016

DOB: date of birth; F: female; HPU: Health Protection Unit; M: male.

The features and functions of the STM are outlined using our example of the fictitious patient Joe Bloggs and fictitious cluster B1363 (Figures 4 and 5).

### Identifying new cases and clusters to review

During a routine local cluster review in the East of England region in August 2017, cluster B1363 was identified to contain a new case, Joe Bloggs, with five cases in total from the Peterborough area. Recent cases (specimen date within the last 3 months) were highlighted in yellow in cluster reports (Figure 4, Panel A) and clusters containing a recent case could be filtered within the cluster report. Alternatively, the cluster report could be downloaded into Microsoft Excel for import into statistical software packages, allowing ad hoc and more sophisticated analysis.

Isolates were assigned to a geographical area, such as the East of England, based on the local laboratory where the isolate originated. Cluster reports were available at a national, regional and local health protection team level. Users could access reports within the STM which showed only clusters and isolates/patients within their jurisdiction.

Demographic, clinical and risk factor information (as entered on ETS at time of notification) could be viewed in the STM for clustered cases where laboratory results had been matched to notifications (Figure 2). This facilitated the review of clustered cases in identifying risk factors for transmission and determining whether cluster investigation was warranted. On review of B1363, it was identified that three of the cases have a history of homelessness (one being Joe Bloggs), four were born in the UK and four had pulmonary TB (Figure 4, Panel A). Based on these risk factors for potential cluster growth associated with a high-risk contextual setting, the cluster investigator recommended that a cluster investigation should be launched and discussed this with the local health protection team, logging the investigation status and actions in the STM (Figure 4, Panel B).

The cluster investigator wanted to see if any other cases existed in the cluster nationally and therefore used the STM search functionality (Figure 5, Panel A). Two searches were available: search by MIRU-VNTR profile and search by cluster number. Each search provided a list of isolates with the strain type and/or cluster number along with a geographical breakdown. The search for cluster B1363 revealed two additional cases in a neighbouring area, one of whom had occurred earlier than any other case in the cluster.

### Cluster investigation data collection tools

To aid cluster investigation, a standard questionnaire was used to obtain social network information including details of the patients' current and past locations of work, worship, socialising or imprisonment, stays at hostels, homeless shelters or hospitals, known exposure to TB and travel abroad or household visitors from abroad [4] (Figure 5, Panel B). Patient-specific questions could also be entered. For B1363 cases, the cluster investigator asked if the Peterborough cases had any links to the neighbouring area of Coventry where the other cases resided. The cluster investigator then submitted the questionnaire request in the system which automatically alerted the case manager to complete. Alternatively, the questionnaire could be downloaded into Microsoft Word and sent by email, which was useful for case managers who were not users of the electronic notification system.

Once received, information collected in the questionnaires was compared by the cluster investigator to identify commonalities between patients and identify possible transmission settings. For fictitious cluster B1363, it was already known by the clinical team from standard contact tracing that Patient One and Patient Two were brothers. Both were homeless and had stayed in the Road One Hostel between January and May 2015, while Joe Bloggs had stayed in this same hostel on and off between November 2014 and April 2015. Before staying in the Road One Hostel, Patient One had been staying with friends in Coventry and regularly visited the Gold Goose Pub while there. This pub was also identified as a regular place of drinking for Other Area Patient A. These epidemiological links are logged in the STM (Figure 5, Panel C).

Patient Three was known to have a household contact, her boyfriend, who previously had active TB and was her likely source of infection but had not been culture-confirmed and therefore did not have a MIRU-VNTR result (thus not appearing in the cluster report). Such links between patients are routinely identified through contact tracing, with households first considered as settings for transmission before investigating and screening contacts from other contextual settings. From the cluster investigation it was also identified that Patient Three's boyfriend was listed as a close contact of Patient One, but the boyfriend had failed to attend screening when invited. Given the likelihood that the boyfriend was part of the cluster, the cluster investigator manually added this epidemiologically linked patient to the cluster (Figure 4, Panel A).

None of the information obtained could link Other Area Patient B to the other cases in the cluster. This cluster investigation identified three possible transmission settings, household(s) and two where awareness raising and screening, beyond screening of close contacts, could be targeted: the hostel in Peterborough and the pub in Coventry.

## Discussion

### Benefits of data integration

Bringing laboratory data into routine surveillance for public health provides effective and timely use of results. This includes using genetic relatedness information to identify possible transmission events and settings for extended contact tracing [7,8], or to refute suspected transmission thereby preventing spending of unnecessary resources. It should be noted that the integration of laboratory and notification data has benefits beyond cluster identification. Other surveillance activities such as routine drug resistance surveillance and zoonotic TB surveillance benefit from the real-time linkage of data, with DST and species results displayed in the notification module of ETS, as well as the STM for clusters. The record linkage also has an important role in monitoring under-notification by identifying how many culture-confirmed patients have not been notified and may not have been started on treatment.

The UK benefits from having a unique national health ID (NHS number), assigned at birth or on registration with primary care services and included in all electronic health records [9]. In recent years, the TB surveillance and control community pushed for better routine use of the NHS number in TB reporting, both in the notification system and on all samples sent to local laboratories and in their referral to reference laboratories. Between 2010 and 2015, 81% of TB notifications had an NHS number entered and 86% of isolate records imported into ETS from reference laboratory information management systems had an NHS number. This ensures that case and laboratory isolate records can be combined automatically at a national level [10]. It facilitates complete and accurate information compiling, with clustering results linked to epidemiological data automatically in the STM for efficient and informed cluster review and investigation to be conducted. Essential to all these activities was the ability to collate and store personal identifiers (names, dates of birth and national identification numbers) nationally, for which a robust and comprehensive information governance structure was in place.

In addition, as the UK has clear data sharing agreements in place, and the two TB data sources used here (notification and laboratory) were collected by one organisation, data linkage was possible. This may not be the case for organisations in other countries where further consideration of data protection issues may be necessary to conduct such data linkage.

### Challenges and limitations

The ability to identify TB clusters relies on the availability of culture confirmation and subsequent genotyping data being available. In the UK between 2010 and 2015, only 61% of TB cases were culture-confirmed, although this proportion was higher at 73% for pulmonary TB cases [1]. Furthermore, 16% of culture-confirmed cases were either not typed or received incomplete typing. Although the system allowed for manual addition of cases to clusters identified through traditional contact tracing and investigation (non-cultured/non-typed), we are unable to quantify the extent to which cluster investigation and the data provided by the system facilitated this identification.

Among cases identified as part of a cluster, data collection benefited from having a structured cluster questionnaire to obtain information on regular places of social contact and interaction, with the ability to add patient-specific questions. This aids the identification of epidemiological links between patients and transmission settings. Unfortunately, while the system allowed the recording of epidemiological links between cases, the use of this functionality was optional and was not always used by local teams investigating the clusters. In addition, the function to record transmission settings may have identified common settings to be followed up during investigation, but was not designed or completed well enough to enable analysis of these data nationally across all clusters.

The cluster questionnaire did not set out how case managers should interview their patients. The questions asked were of a personal nature and patients may feel these were intrusive; this was particularly true if the social network included drug dealing and use. Often the information received from cluster questionnaires was limited, but it was not known whether this was due to patients’ reluctance to provide information or if the questions were not posed in a way to facilitate the patients’ recall. Training and decisions about how to phrase these questions to patients in order to get the most reliable information should be considered, as has been done by others [11]. However, there was also the awareness that retrospective data collection had its limitations. In our fictitious scenario, transmission settings were identified, but at least 2 years had passed since the transmission occurred. Consequently, any screening activities would have limited benefit, and this was often the case in real clusters and transmission settings. Therefore, there is currently a desire to conduct prospective data collection on social networks at the time of notification as soon as two or more patients are molecularly linked, allowing earlier review of information. This should facilitate more timely and appropriate public health action once a cluster has been identified. Conversely, a small proportion of patients will be part of a cluster, and even fewer will be part of a cluster of public health importance; therefore, a large volume of unused data would need to be collected and inputted by case managers.

Unfortunately, as the evaluation of the STS reported [6], the use of strain typing data and resulting public health action was limited by the fact that the STM was not available at the inception of strain typing. The evaluation concluded that there was no evidence that cluster investigation was a cost-effective TB control method, recommending that routine systematic cluster investigations should be terminated and instead initiated in response to local demand. This finding may have subsequently affected the level of user engagement once the STM was fully implemented. Early consideration of software visualisation and logging (cluster review and investigation findings) systems remain an essential component of molecular technology roll-out.

### Future opportunities

Genotyping results have a clear role in TB control, and the availability of these results and integration into routine surveillance activity are important. This has been exemplified previously by those working in TB surveillance and control in United States in their overview of the database created there to identify TB clusters [12]. What remains essential is that these systems have a design led by public health.

As the UK moves to replace MIRU-VNTR with WGS, more complex data displays are necessary. This includes phylogenetic trees and matrix tables to quantify and visualise relatedness as sequencing data cannot be visualised as simply as strain typing data, i.e. as either the same 24 digit strain type or not. In the UK, the integration of such functionality into ETS was not possible given the age of the system (initially developed in 2008); therefore, a new system is required. A replacement surveillance system is planned but given the immediate roll-out of WGS, an interim solution to combine patient data and sequencing has to be developed. Interim and long-term solutions present the possibility of creating a system which builds on the successes of the STM in terms of data integration and linkage. This involves bringing together demographic, clinical, risk factor and social network information with molecular clustering results. In addition, functionality to log cluster review and investigation action and findings including epidemiological links and/or transmission settings is needed, as well as functionality to add non-culture confirmed, therefore not molecularly linked, cases. From use of the STM, we have identified additional features which could enhance and streamline cluster review and investigation processes, which are currently performed manually outside of the STM: firstly, the ability to create outputs such as timelines symbolising each time a patient is culture- or sputum-positive and therefore at risk of transmitting, secondly, improved geographical assignment of isolates and/or patients based on postcodes rather than source laboratories, as often the specimen referral pathways do not correlated with geographical location of patient residence or treatment; thirdly, the importance of local users having access to cluster results from across the country rather than only patients from their jurisdiction to allow efficient cross-boundary public health action.

While this all presents challenges, there are more immediate benefits to be gained, such as the short time it takes to obtain sequencing results [13] compared with the time for strain typing, which we identified to be a median of more than 2 months from specimen date to receipt in the surveillance system. The timeliness of relatedness data along with prospective social network data collection should have an impact on improving TB control in England. In addition, there are exciting opportunities for pan-European identification of cluster and strain relatedness if sequencing data are held in a centralised system. A pilot project focusing on TB cases with multidrug resistance is underway [14].
